# Meta-Analysis of the Association between Transforming Growth Factor-Beta Polymorphisms and Complications of Coronary Heart Disease

**DOI:** 10.1371/journal.pone.0037878

**Published:** 2012-05-25

**Authors:** Dylan R. Morris, Joseph V. Moxon, Erik Biros, Smriti M. Krishna, Jonathan Golledge

**Affiliations:** Vascular Biology Unit, School of Medicine and Dentistry, James Cook University, Townsville, Australia; FuWai Hospital - Chinese Academy of Medical Sciences, China

## Abstract

**Objective:**

To investigate the association between common transforming growth factor beta (*TGF-β*) single nucleotide polymorphisms (SNP) and significant complications of coronary heart disease (CHD).

**Method:**

We performed a meta-analysis of published case-control studies assessing the association of *TGF-β* SNPs with a range of CHD complications. A random effects model was used to calculate odds ratios and confidence intervals. Analyses were conducted for additive, dominant and recessive modes of inheritance.

**Results:**

Six studies involving 5535 cases and 2970 controls examining the association of common SNPs in *TGF-β1* with CHD were identified. Applying a dominant model of inheritance, three *TGF-β1* SNPs were significantly associated with CHD complications: The T alleles of *rs1800469* (OR = 1.125, 95% CI 1.016–1.247, p = 0.031) and *rs1800470* (OR = 1.146, 95% CI 1.026–1.279, p = 0.021); and the C allele of *rs1800471* (OR = 1.207, 95% CI 1.037–1.406, p = 0.021).

**Conclusion:**

This meta-analysis suggests that common genetic polymorphisms in *TGF-β1* are associated with complications of CHD.

## Introduction

Coronary heart disease (CHD) is the leading cause of mortality in the western world. In the U.S.A. CHD is responsible for 1 in every 6 deaths and is estimated to cost over US$177.5 billion per annum. [1] Early stage CHD is asymptomatic, but advanced disease is associated with severe complications including myocardial infarction, heart failure, unstable angina and sudden cardiac arrest. [2] Well defined risk factors for CHD include smoking, diabetes, hypertension, dyslipidaemia, male gender and age. A family history of CHD also confers significant risk, demonstrated by an eightfold increase in monozygotic twins and a fourfold increase in dizygotic twins of affected individuals. [3] The genetic mechanisms underlying CHD predisposition are poorly understood despite recent findings from genome-wide association studies (GWAS) (discussed in detail by Patel *et al.* (2011)). [4].

Data from independent gene studies suggest a putative role for *TGF-β* signalling in CHD. [5], 6 A range of potentially athero-protective roles have been described for the *TGF-β* isoforms, favouring a stable fibrous plaque over an unstable inflammatory plaque. In vitro studies have shown *TGF-β1* to upregulate extracellular matrix (ECM) production, inhibit vascular smooth muscle cell proliferation and impair division and migration of endothelial cells. [7] Additionally, *TGF-β1* modulates the local inflammatory response by down-regulating expression of endothelial adhesion molecules, inhibiting foam cell formation and impeding proliferation of CD4 and CD8 T lymphocytes. [7] Dysregulation of *TGF-β* signalling resulting from genetic mutations, particularly single nucleotide polymorphisms (SNPs), within components of the *TGF-β* signalling pathway have been linked to an increased risk of developing common vascular diseases. 8,9 Some but not all studies suggest a positive association between CHD and several SNPs within genes encoding *TGF-β* cytokines. [5], [6], [10], [11], [12], [13] Given the current discordance in published studies, the involvement of *TGF-β* SNPs in CHD pathogenesis remains unclear. Accordingly, we conducted a meta-analysis combining publically available data with the aim of clarifying whether *TGF-β* SNPs are associated with clinically significant complications of CHD.

## Methods

### Search Strategy

A search strategy was devised to identify published studies assessing the association of *TGF-β1*, *TGF-β2* and *TGF-β3* SNPs with end-stage complications of CHD. The search followed the guidelines of the 2009 preferred reporting items for systematic reviews and meta-analysis (PRISMA) statement and was undertaken in two stages. [14] Initially, the MEDLINE database was searched using the terms “transforming growth factor” or “TGF” and “SNP” or “polymorphism” and “coronary heart disease”, limits human. Focused searches were also undertaken as above, including “myocardial infarction”, “unstable angina”, “heart failure” or “sudden cardiac arrest” as specific complications of CHD. Hand-searching of references and use of the related articles function in Pubmed was performed to identify any additional studies. Finally, following the recommendations of Stern and Simes (1997), we minimized the possibility of publication bias in our meta-analysis by searching the National Institute of Health clinical trials registry (http://clinicaltrials.gov/) using original search terms to identify unpublished studies eligible for inclusion in the current analysis. These searches were restricted to studies that started before 2010 to avoid the problem of the delayed publication of studies with negative results and included both large and small scale investigations. [15].

**Table 1:** Characteristics of 6 case-control studies included in the meta-analysis.

		*Cambien et al. * [*20*]	*Syrris et al. * [*13*]	*Yokota et al. * [*18*]	*Holweg et al. * [*12*]	*Koch et al. * [*19*]	*Crobu et al. * [*11*]
Year		*1996*	*1998*	*2000*	*2001*	*2006*	*2008*
Location		Northern Ireland; France	London and Sheffield, England	Nagoya, Japan	Rotterdam	Munich	Turin, Italy
CHD presentation		MI	Angiographically-proven CHD†	MI	End stage heart failure	MI	MI
Diagnosis of cases		Satisfy WHO criteria for diagnosis of MI	Coronary angiography	ECG, blood tests, coronary angiography	N.S.	ECG, coronary angiography	Satisfy WHO criteria for diagnosis of MI
Screening of controls		Questionnaire of CHDrisk factors	Coronary angiography	ECG & exercise stresstest	N.S.	Coronaryangiography	None
Participants	Cases	563	655	315	144	3657	201
	Controls	629	244	591	94	1211	201
Age (years, mean and std. dev.)	Cases	54.2±8.1	60.3±9.9	54.9±5.9	50.8±7.7	64.0+12.0	40±4.0
	Controls	52.8±8.4	56.9±11.2	57.0±6.1	36.7±10.3	60.3±11.9	40±4.0
Males (%)	Cases	100	79.5*	74.3*	93.8*	75.8*	N.S. ‡
	Controls	100	31.1	48.9	52.1	50.6	N.S. ‡
Smokers (%)	Cases	N.S.	43.4*	63.4*	N.S.	50.6*	79.1*
	Controls	N.S.	26.4	47.7	N.S.	15.2	59.7
Hypertension (%)	Cases	N.S.	30.7	N.S.	N.S.	61.4*	33.9*
	Controls	N.S.	27.0	N.S.	N.S.	48.6	16.3
Diabetes (%)	Cases	N.S.	6.3	20.6	N.S.	20.6*	N.S.
	Controls	N.S.	5.2	22.7	N.S.	5.4	N.S.
Dyslipidaemia (%)	Cases	N.S.	N.S.	56.2	N.S.	50.6	54.9*
	Controls	N.S.	N.S.	53.8	N.S.	49.7	35.7

MI, myocardial infarction; CHD, coronary heart disease; WHO, World Health Organization; N.S., not stated.

* Indicates significant difference in the occurrence of CHD risk factor between case and control populations.

† Patients were recruited following angiography demonstrating greater than 30% reduction in the diameter of one or more coronary arteries.

‡ Author did not provide relevant data, but stated that cases and controls were matched for age and sex.

### Study Selection

Studies included were case-control investigations comparing the association of *TGF-β1*, *TGF-β2* and *TGF-β3* SNPs with clinical endpoints of CHD. Studies in which subjects did not have documented symptoms and diagnostic evidence of CHD were excluded. Studies which included cases with mixed types of heart disease, such as congenital heart disease, were excluded. Animal studies were excluded. Abstracts were initially screened by the primary author (DM). If a decision could not be made regarding inclusion, the full text of the article was examined.

**Figure 1:**
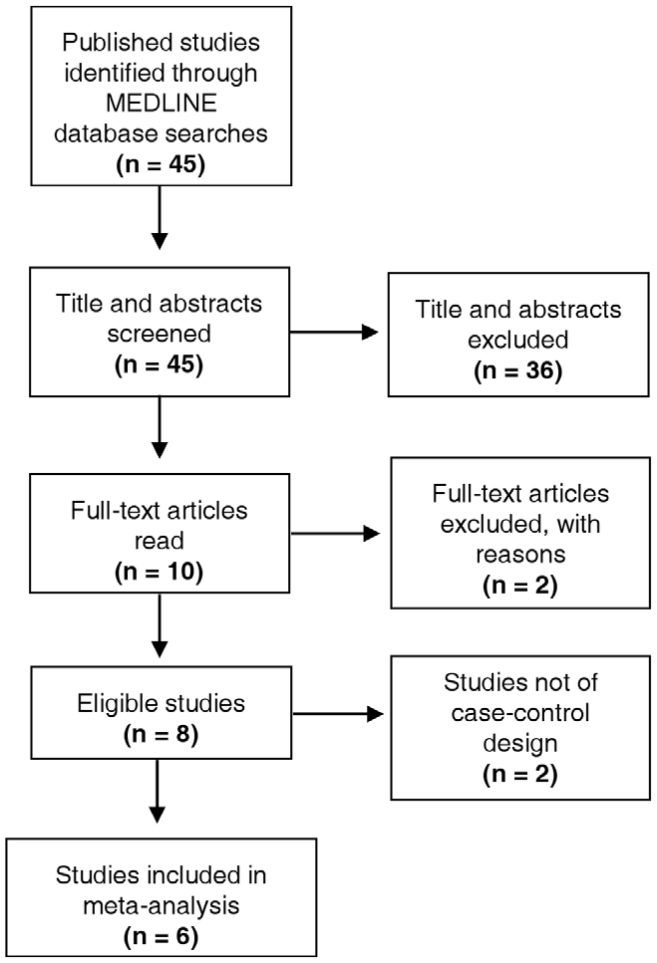
Flow diagram showing studies included. 45 published studies assessing the association of *TGF-β* SNPs and CHD were identified by searching the MEDLINE database. Appraisal of the abstracts identified 10 articles eligible for full-text appraisal. From these, a further 2 articles were excluded, leaving 8 articles for inclusion in the systematic review. Six of these studies adopted a case-control design, and data from these were extracted for further assessment in the meta-analysis.

### Data Extraction

The following data were independently extracted from selected studies by two authors (DM & JM) using a standardised data extraction form: 1) study design; 2) number of participants in case and control groups; 3) patient characteristics of each group; 4) method of screening for CHD; 5) SNP(s) assessed; and 6) odds ratio (OR) and 95% confidence interval for association with CHD. Limitations or biases stated by the authors of each study were also recorded. SNP annotation was standardised using RS numbers as stated by the publishing authors. If this detail was not provided, RS numbers were assigned using the dbSNP resource on the NCBI server where possible. [16] After completion of the data extraction, the two reviewers compared their findings and resolved any disagreements at a consensus meeting. The data extraction prototol utilised in this study, and proof of compliance with PRISMA guidelines are provided as Text S1 and Table S1 respectively.

### Quantitative Data Synthesis

Combined unadjusted ORs for the association of *TGF-β* SNPs with CHD complications were generated using additive, dominant and recessive inheritance under a random effects model. For the rs1800469, rs1800470 and rs1800471 SNPs publication bias was assessed using funnel plots. Heterogeneity analysis was performed using the Cochran Q test and I2 index. Sensitivity analyses were performed using the one-study remove approach to assess the impact of each study on the combined effect as previously described. [9] All computations were performed using the Comprehensive Meta Analysis v2 software package (Biostat, Inc.).

**Table 2:** Meta-analysis of published associations between *TGF-β1* SNPs and complications of CHD.

SNP	Population	Cases	Controls	OR*	95% CI	p value
rs1800468^∼^	*Syrris et al. * [*13*]	655	244	1.246	0.826–1.80	0.294
	*Cambien et al. * [*20*]	563	629	1.084	0.792–1.482	0.615
	*Crobu et al. * [*11*]	201	201	0.756	0.434–1.319	0.325
	* **Combined**	**1419**	**1074**	**1.064**	**0.846–1.339**	**0.346**
rs1800469^±^	*Syrris et al. * [*13*]	655	244	1.215	0.905–1.631	0.194
	*Cambien et al. * [*20*]	563	629	0.967	0.768–1.217	0.776
	*Koch et al. * [*19*] ‡	3657	1211	1.145	1.005–1.304	0.042
	*Crobu et al. * [*11*]	201	201	1.322	0.880–1.987	0.179
	* **Combined**	**5076**	**2285**	**1.125**	**1.016–1.247**	**0.031**
rs1800470^§^	*Syrris et al. * [*13*]	655	244	1.226	0.908–1.654	0.183
	*Cambien et al. * [*20*]	563	629	1.175	0.924–1.465	0.188
	*Koch et al. * [*19*] ‡	3657	1211	1.193	1.042–1.365	0.01
	*Crobu et al. * [*11*]	201	201	1.388	0.907–2.123	0.131
	*Yokota et al. * [*18*] †	315	591	0.856	0.629–1.164	0.322
	*Holweg et al. * [*12*]	144	94	0.909	0.535–1.544	0.724
	* **Combined**	**5535**	**2970**	**1.146**	**1.026–1.279**	**0.021**
rs1800471^¤^	*Syrris et al. * [*13*]	655	244	1.24	0.800–1.923	0.337
	*Cambien et al. * [*20*]	563	629	1.404	1.022–1.927	0.036
	*Koch et al. * [*19*] ‡	3657	1211	1.159	0.953–1.409	0.14
	*Holweg et al. * [*12*]	144	94	0.899	0.438–1.848	0.772
	* **Combined**	**5019**	**2178**	**1.207**	**1.037–1.406**	**0.021**
rs1800472°	*Syrris et al. * [*13*]	655	244	1.796	0.784–4.116	0.166
	*Cambien et al. * [*20*]	563	629	0.638	0.389–1.044	0.074
	*Koch et al. * [*19*] ‡	3657	1211	1.033	0.791–1.349	0.812
	* **Combined**	**4875**	**2084**	**0.978**	**0.622–1.536**	**0.397**

OR, odds ratio; CI, 95% confidence intervals.

∼Rs1800468: promoter, in terms of minor A allele; ^±^Rs1800469: promoter, in terms of minor T allele; ^§^Rs1800470: coding sequence, in terms of minor T allele; ¤Rs1800471: coding sequence, in terms of minor C allele; °Rs1800472: coding sequence, in terms of minor T allele.

* Combined, indicates meta-analysis data calculated for a Random Effects model, assuming dominant inheritance. Data for additive and recessive models displayed in Table S1.

† Odds ratio given for combined male and female population, not just male population as provided by author.

‡ Odds ratio given in terms of minor allele, not major allele as stated in publication.

## Results

### Flow of Included Studies

From 45 publications identified by initial data searches, 10 studies fulfilled inclusion criteria for the systematic review and were selected for further assessment (Figure 1). The full texts of these 10 studies were evaluated, after which 2 articles were excluded. One study was not included as it assessed patients only with end stage renal disease. [17] Furthermore, the case population included patients with non-cardiac atherosclerotic disease. [17] The second study assessed the prevalence of a selected *TGF-β2* SNP in CHD patients, however, the SNP that was assessed was not named. [10] The remaining 8 studies focussed solely on *TGF-β1* SNPs in cases and controls (Table 1). Furthermore the study of Sie *et al.* (2006) utilised a prospective cohort design and that of Manginas *et al. (2008)* did not include a control group with which to compare genotype distributions. [5], [6] Accordingly, these two studies were also excluded, leaving 6 independent case-control investigations in the current meta-analysis (Figure 1).

**Table 3:** Heterogeneity analysis of combined effect size.

		Additive model				Dominant Model				Recessive Model			
SNP	Genotype	Q	df (Q)	P	I^2^	Q	df (Q)	P	I^2^	Q	df (Q)	P	I^2^
rs1800469	CC	–	–	–	–	–	–	–	–	–	–	–	–
	CT	2.351	3	0.503	0.000	2.595	3	0.458	0.000	–	–	–	–
	TT	4.017	3	0.260	0.253					4.634	3	0.201	0.353
rs1800470	TT	–	–	–	–	–	–	–	–	–	–	–	–
	TC	0.767	5	0.979	0.000	5.532	5	0.354	0.096	-	–	–	–
	CC	25.854	5	<0.001	0.807					31.145	5	<0.001	0.839
rs1800471	GG	–	–	–	–	–	–	–	–	–	–	–	–
	GC	1.870	3	0.600	0.000	1.694	3	0.638	0.000	–	–	–	–
	CC	2.747	3	0.432	0.000					2.731	3	0.435	0.000

Q, Cochran Q test; df, degrees of freedom; I^2^, I^2^ index.

### Study Characteristics

A collective total of 8,505 patients were included across the 6 studies (cases n = 5535, controls n = 2970). Five investigations were conducted in patients from a Western European background and one study was conducted using Japanese recruits. [18] Population sizes of the independent studies ranged from 238 to 4868 participants. Cases included patients with myocardial infarction (4 studies), ischemic heart failure (1 study) or patients with angiographically-proven CHD (1 study). [13] Three studies utilised coronary angiography to confirm a positive diagnosis of CHD whereas two studies relied on the World Health Organisation diagnostic criteria for MI, employing clinical history, ECG and cardiac biomarkers. Likewise, there was also considerable inter-study variation in screening of controls, with only two studies utilising coronary angiography to confirm a negative diagnosis of CHD. [13], [19] Common CHD risk factors for cases and controls are shown in Table 1. With the exception of age and gender, studies did not match cases and controls for conventional CHD risk factors. Moreover, the distribution of risk factors was not uniformly reported across studies and two studies failed to document the occurrence of environmental risk factors. [12], [19] Reported data suggested that CHD populations demonstrated a higher prevalence of hypertension, smoking, diabetes and dyslipidaemia. Only two investigations sub-divided population groups by sex. [18], [19].

**Figure 2:**
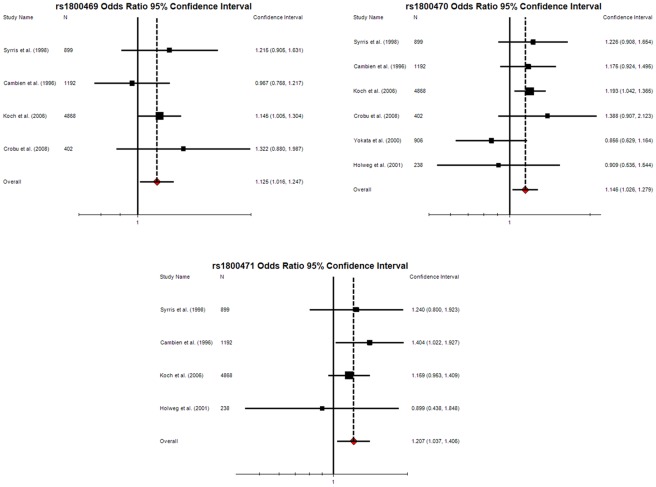
Forest plots detailing unadjusted odds ratios and 95% confidence intervals for the association between TGFβ1 rs1800469, rs1800470 and rs1800471 with end-stage CHD calculated using the dominant model of inheritance. The dotted line represents overall odds ratio calculated in the current meta-analysis for each SNP.

**Table 4:** Leave-one-out sensitivity analysis of combined effect size.

			Additive model			Dominant model			Recessive model		
SNP	Study removed	Genotype	OR	95% CI	P	OR	95% CI	P	OR	95% CI	P
rs1800469	*Syrris et al. * [*13*]	CC	–	–	–	–	–	–	–	–	–
		CT	1.105	0.977–1.249	>0.05	1.110	0.978–1.259	>0.05	–	–	–
		TT	1.190	0.890–1.591	>0.05				1.160	0.862–1.562	>0.05
	*Cambien et al. * [*20*]	CC	–	–	–	–	–	–	–	–	–
		CT	1.168	1.035–1.319	<0.05	1.168	1.042–1.310	<0.05	–	–	–
		TT	1.271	0.917–1.762	>0.05				1.202	0.852–1.697	>0.05
	*Koch et al. * [*19*]	CC	–	–	–	–	–	–	–	–	–
		CT	1.052	0.883–1.253	>0.05	1.106	0.918–1.333	>0.05	–	–	–
		TT	1.301	0.922–1.837	>0.05				1.263	0.932–1.712	>0.05
	*Crobu et al. * [*11*]	CC	–	–	–	–	–	–	–	–	–
		CT	1.113	0.979–1.266	>0.05	1.113	1.001–1.237	<0.05	–	–	–
		TT	1.085	0.910–1.294	>0.05				1.027	0.869–1.214	>0.05
rs1800470	*Syrris et al. * [*13*]	TT	–	–	–	–	–	–	–	–	–
		TC	1.149	1.029–1.283	<0.05	1.123	0.978–1.291	>0.05	–	–	–
		CC	1.028	0.656–1.611	>0.05				0.969	0.625–1.502	>0.05
	*Cambien et al. * [*20*]	TT	–	–	–	–	–	–	–	–	–
		TC	1.151	1.027–1.291	<0.05	1.126	0.968–1.309	>0.05	–	–	–
		CC	1.047	0.639–1.714	>0.05				0.986	0.608–1.601	>0.05
	*Koch et al. * [*19*]	TT	–	–	–	–	–	–	–	–	–
		TC	1.119	0.961–1.304	>0.05	1.104	0.936–1.303	>0.05			
		CC	1.041	0.606–1.788	>0.05				0.985	0.577–1.679	>0.05
	*Crobu et al. * [*11*]	TT	–	–	–	–	–	–	–	–	–
		TC	1.156	1.039–1.287	<0.05	1.127	0.999–1.270	>0.05	–	–	–
		CC	0.950	0.651–1.386	>0.05				0.882	0.615–1.264	>0.05
	*Yokota et al. * [*18*]	TT	–	–	–	–	–	–	–	–	–
		TC	1.166	1.044–1.301	<0.05	1.192	1.074–1.322	<0.05	–	–	–
		CC	1.298	1.010–1.667	<0.05				1.210	0.937–1.563	>0.05
	*Holweg et al. * [*12*]	TT	–	–	–	–	–	–	–	–	–
		TC	1.158	1.042–1.288	<0.05	1.156	1.028–1.300	<0.05	–	–	–
		CC	1.156	0.777–1.719	>0.05				1.076	0.726–1.595	>0.05
rs1800471	*Syrris et al. * [*13*]	GG	–	–	–	–	–	–	–	–	–
		GC	1.182	1.002–1.395	<0.05	1.203	1.023–1.415	<0.05	–	–	–
		CC	1.828	0.674–4.954	>0.05				1.787	0.663–4.821	>0.05
	*Cambien et al. * [*20*]	GG	–	–	–	–	–	–	–	–	–
		GC	1.144	0.959–1.366	>0.05	1.154	0.970–1.373	>0.05	–	–	–
		CC	1.224	0.554–2.701	>0.05				1.205	0.546–2.659	>0.05
	*Koch et al. * [*19*]	GG	–	–	–	–	–	–	–	–	–
		GC	1.221	0.955–1.561	>0.05	1.285	1.009–1.637	<0.05	–	–	–
		CC	3.667	1.033–13.012	<0.05				3.576	1.008–12.680	<0.05
	*Holweg et al. * [*12*]	GG	–	–	–	–	–	–	–	–	–
		GC	1.210	1.032–1.418	<0.05	1.224	1.048–1.430	<0.05	–	–	–
		CC	1.553	0.676–3.570	>0.05				1.490	0.669–3.320	>0.05

OR, odds ratio; 95% CI, 95% confidence intervals. Analysis was performed using a random effects model.

Five TGF-β1 SNPs (rs1800468, rs1800469, rs1800471, rs1800472, rs1800470) were investigated in the included studies. The rs1800468 (G-800A) and rs1800469 (C-509T) SNPs are located in the gene promoter, whereas the rs1800470 (Leu10→Pro), rs1800471 (Arg25→Pro) and rs1800472 (Thr263→Ile) polymorphisms are located in coding regions (Table 2). Cambien et al. (1996) assessed two additional SNPs which were not included in the meta-analysis. [20] The rs1800820 SNP (C-988A) was present in only 2 of the 1192 participants, and the +72C SNP was reported to be in complete linkage with rs1800471, but could not be identified on dbSNP. [20] None of the 6 studies found any associations between rs1800468 or rs1800472 and end-stage CHD. [6], [11], In contrast, CHD-associated MI was linked to the minor T alleles of rs1800469 and rs1800470 by Koch et al. (2006) (odds ratios 1.14, 95% CI 1.00–1.30, P  = 0.042) and Yokata et al. (2000) (odds ratio 3.5, 95% CI 2.0–6.3, P<0.0001) respectively, although this conflicts with findings from other authors. [5], [6], [11], [12], [13], [18], [19], [20] Similarly, Cambien et al. (1996) found that rs1800471 was associated with CHD complications (odds ratio 1.40, 95% CI 1.02–1.92, P  = 0.036), however this was not supported by data generated by subsequent investigations into this polymorphism. [5], [6], [12], [13], [19], [20] It is important to note that Koch et al. (2006) and Yokata et al. (2000) separated their results by sex, and found positive associations only in the male populations. [18], [19] The association identified by Cambien et al. (1996) was also restricted to a male population, as this study did not include female participants. [20].

### Quantitative Data Synthesis

The association between reported *TGF-β1* SNPs and complications of CHD was assessed using additive, dominant and recessive models of inheritance (Table 2 and Table S2). Analyses revealed no significant association of the *TGF-β1* rs1800468 and rs1800472 under any model, and these SNPs were not further investigated. Heterogeneity assessments for rs1800469, rs1800470 and rs1800471 demonstrated significant inter-study variation, particularly for rs1800470, under additive and recessive models (Table 3 and Table S2), thus data from these models were disregarded. In contrast, no heterogeneity was observed for calculations made using the dominant model of inheritance suggesting that conclusions made applying this algorithm were relatively robust. Findings from the dominant model of inheritance were therefore selected for further assessment. Significant positive associations were observed between CHD complications and the minor alleles of rs1800469 (OR 1.125, 95% CI 1.016–1.247, P  = 0.031), rs1800470 (OR 1.146, 95% CI 1.026–1.279, P  = 0.021) and rs1800471 (OR 1.207, 95% CI 1.037–1.406, P  = 0.021; Table 2). As expected, odds ratios generated by the current meta-analysis differed from findings of previous studies (Figure 2). Funnel plots suggested no publication bias was observed for these SNPs (Figure S1). Sensitivity analyses confirmed that multiple studies contributed to the observed significant associations between rs1800469, rs1800470 and end-stage CHD under a dominant model of inheritance (Table 4). In contrast, the significant association between rs1800471 and CHD end points was only lost if the individual study by Cambien *et al.* (1996) was removed. [20].

## Discussion

The association between SNPs of the *TGF-β1* cytokine and clinically significant cardiac events was previously unclear due to conflicting data generated by a range of independent studies. To clarify this we conducted a meta-analysis of publically available data, confirming significant associations between the minor alleles of *TGF-β1 rs1800469*, *rs1800470* and *rs1800471* with CHD when applying a dominant model of inheritance. Odds ratios generated for these SNPs were relatively low (1.125 – 1.207), as expected for a multifactorial disease such as CHD.

Odds ratios generated by our meta-analysis for *TGF-β1 rs1800469*, *rs1800470* and *rs1800471* are lower than previously reported, [18], [19], [20] possibly reflecting the larger, more genetically diverse population analysed in this study. Odds ratios may also have been lowered as we assessed patient populations of mixed gender. Previously Cambien *et al.* (1996), Yokata *et al.* (2000) and Koch *et al.* (2006) independently identified positive associations between these SNPs and end-stage CHD in their male recruits only. [18], [19], [20] These associations were not observed in cohorts comprising both male and female subjects. [11], [12], [13] This tentatively suggests that these SNPs may be more relevant to CHD progression in male carriers although this hypothesis could not be assessed in the current study as sex-specific data were not provided by all investigators.

Mechanisms underpinning the positive associations of *TGF-β1 rs1800649*, *rs1800470* and *rs1800471* with development of end-stage CHD remain unclear. These SNPs are located within functional regions of the *TGF-β1* gene and it is therefore plausible to suggest that they may directly impact on *TGF-β1* signalling. For example, *rs1800469* is situated within the promoter region of the *TGF-β1* gene and has been reported to alter secretion of *TGF-β1* in vitro. [21] Interestingly, Yokota *et al.* (2000) reported reduced serum concentrations of *TGF-β1* in subjects carrying the *TC* or *TT* genotype *rs1800469* compared to the *CC* genotype. [18] No significant difference in circulating *TGF-β1* was observed between case (myocardial infarction) or control recruits of the same genotype, suggesting that altered circulating levels of *TGF-β1* are not the principal cause of CHD complications in this population. *Rs1800470* and *rs1800471* are both located within exons, resulting in amino acid changes of *Leu10Pro* and *Arg25Pro* respectively. Cox *et al.* (2007) reported that the proline substitution at codon 10 is associated with higher circulating concentrations of acid-activatable *TGF-β1* and increased secretion in vitro. [22] This may be a result of altered intracellular trafficking, or increased transcription. Substitution of proline residues are also likely to alter the chemical properties and tertiary structure of *TGF-β1* protein, potentially interfering with receptor interactions. However, these effects are yet to be described.

### Limitations

This study has several limitations. Firstly, only 6 published studies fulfilled our selection criteria, thus the meta-analysis was restricted to a relatively small patient population primarily comprising subjects from Western Europe. Importantly, these studies focused solely on *TGF-β1* SNPs and we were therefore unable to investigate the contribution of *TGF-β2* or *TGF-β3* polymorphisms to the development of end-stage CHD. Secondly, we were unable to adjust the meta-analysis to correct for common cardiovascular risk factors (e.g. gender or smoking) as these were not uniformly reported in the independent investigations and could not be easily obtained. This was further complicated by significant variation in clinical definitions of hypertension and dyslipidaemia. [13] It is therefore possible that calculated ORs and P values are over-estimates of the true biological situation. Thirdly, significant heterogeneity was observed between studies suggesting that these data could not be investigated using the additive and recessive models of inheritance. Similarly, we noted significant inter-study variation in methods employed to screen control populations. Only 2 of the analysed studies provided a definitive negative CHD diagnosis in controls via coronary angiography, [13], [19] whereas other studies employed non-specific methodologies, such as electrocardiography, or a complete lack of screening. [11], [12], [18], [20] Consequently, there is potential for false negative diagnoses in some studies, and it is feasible to suggest that control subjects may not truly represent healthy (CHD-free) individuals. Finally, data collected from patients presenting with a range of cardiovascular complications were appraised in this analysis, thereby assuming that similar pathological mechanisms underpin distinct CHD endpoints. Subsequently, we are unable to suggest specific contributions of *TGF-β1* SNPs to the development of discrete events such as myocardial infarction and end stage heart failure.

The authors attempted to include SNPs related to the *TGF-β* genes from previous GWAS in the present meta-analysis. Prior to data extraction, the Catalogue of Published Genome-Wide Association Studies (genome.gov) was searched and 16 CHD GWAS were identified. [23] The full text and supplementary data of these studies were hand-searched by the primary author. The risk alleles identified in this meta-analysis of *TGF-β* gene studies have not been highlighted in recent GWAS focused on the presence or complications of CHD. The reasons for differences between findings of candidate gene and genome wide association studies are currently unexplained. Some possible reasons include: risk alleles only acting in certain populations due to variation in other genetic factors or environmental exposure, false discovery, confounding effects of combining samples and data from very different studies where information is collected under different definitions, variation in demographic profile of control populations, and heterogeneity of data collection methods across studies.

### Conclusion

In conclusion, we confirm significant positive associations between the *TGF-β1* SNPs *rs1800469*, *rs1800470* and *rs1800471* and complications of CHD. Further work is required to determine the clinical relevance of these findings.

## Supporting Information

Figure S1 Funnel plots detailing publication bias for TGF-β1 SNPs rs1800469, rs1800470 and rs1800471 using the dominant model of inheritance.
(TIF)

Table S1 Checklist to confirm compliance with PRISMA guidelines for systematic reviews and meta-analyses.
(DOC)

Table S2 Meta-analysis of published associations between TGF-β1 SNPs and CHD complications under additive, dominant and recessive models of inheritance.
(DOC)

Text S1 Protocol used for systematic data collection from eligible articles.
(DOC)
