# Supplementary figures and images for: Meta-Analysis of the Association between Transforming Growth Factor-Beta Polymorphisms and Complications of Coronary Heart Disease
Source: PLoS One. 2012 May 25;7(5):e37878. doi: 10.1371/journal.pone.0037878 (PMC3360665; doi:10.1371/journal.pone.0037878)

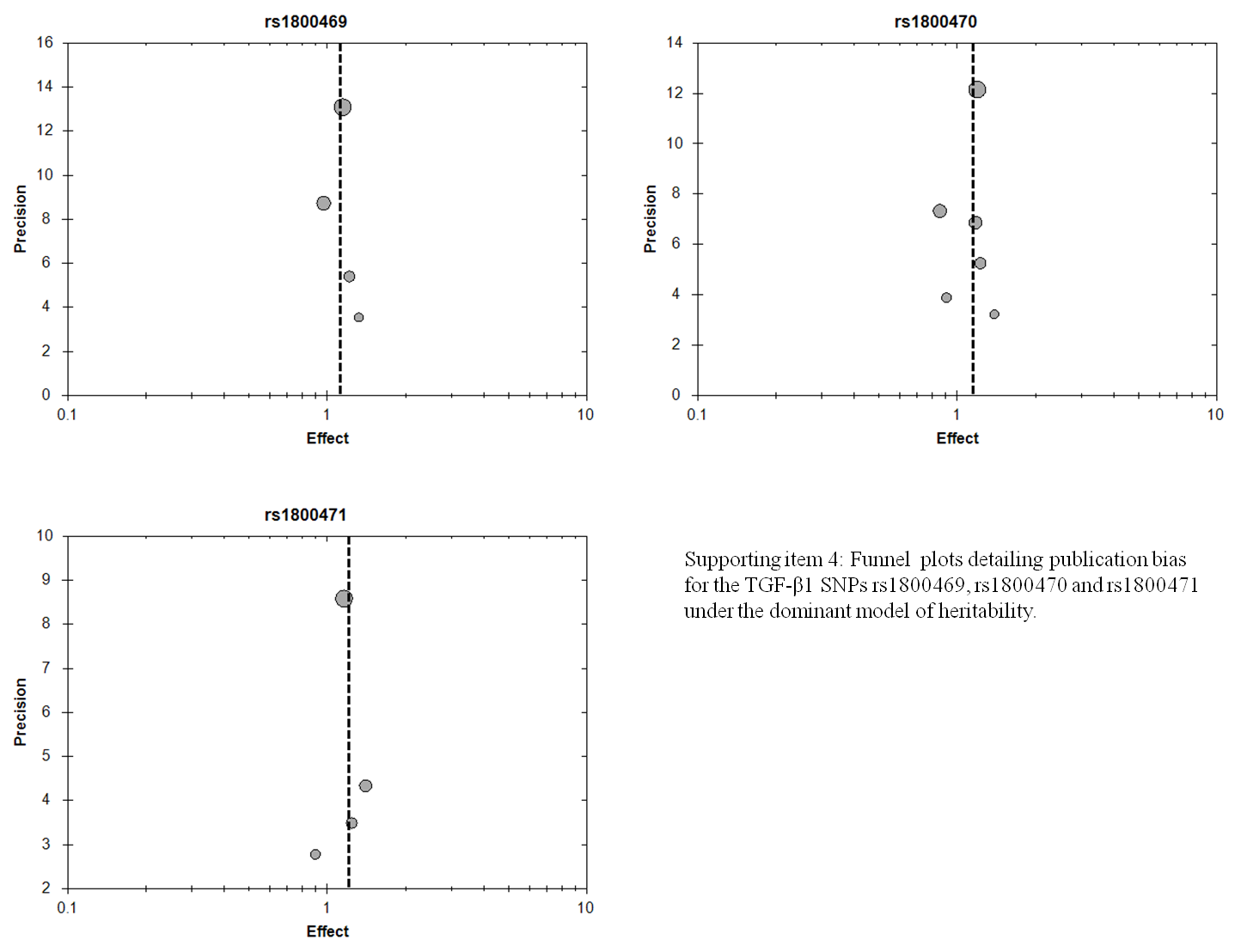

Supplement: Figure S1 — Funnel plots detailing publication bias for TGF-β1 SNPs rs1800469, rs1800470 and rs1800471 using the dominant model of inheritance. (TIF) [file pone.0037878.s001.tif]
