# Supplementary material for: Meta-Analysis of the Association between Transforming Growth Factor-Beta Polymorphisms and Complications of Coronary Heart Disease
Source: PLoS One. 2012 May 25;7(5):e37878. doi: 10.1371/journal.pone.0037878 (PMC3360665; doi:10.1371/journal.pone.0037878)
Supplement: Table S2 — Meta-analysis of published associations between TGF-β1 SNPs and CHD complications under additive, dominant and recessive models of inheritance. (DOC) [file pone.0037878.s003.doc]

**Supporting item 3: Meta-analysis of published associations between TGF-β1 SNPs and CHD complications using additive, dominant and recessive models of inheritance.**

| **SNP** | **Study** | **N** | | **Genotype** | **Additive Model** | | | **Dominant Model** | | | **Recessive Model** | | |
| --- | --- | --- | --- | --- | --- | --- | --- | --- | --- | --- | --- | --- | --- |
|  |  | **Cases** | **Controls** |  | **OR** | **95% CI** | **P** | **OR** | **95% CI** | **P** | **OR** | **95% CI** | **P** |
| rs1800468 | *Syrris et al.*[*^13^*](#_ENREF_13) | 541 | 207 | GG | - | - | - | - | - | - | - | - | - |
|  |  | 110 | 34 | GA | 1.238 | 0.816-1.878 | 0.315 | 1.246 | 0.826-1.80 | 0.294 | - | - | - |
|  |  | 4 | 1 | AA | 1.533 | 0.170-13.799 | 0.703 |  |  |  | 1.481 | 0.165-13.315 | 0.726 |
|  | *Cambien et al.*[*^20^*](#_ENREF_20) | 472 | 534 | GG | - | - | - | - | - | - | - | - | - |
|  |  | 88 | 89 | GA | 1.119 | 0.813-1.540 | 0.492 | 1.084 | 0.792-1.482 | 0.615 | - | - | - |
|  |  | 3 | 6 | AA | 0.566 | 0.141-2.274 | 0.422 |  |  |  | 0.556 | 0.138-2.235 | 0.408 |
|  | *Crobu et al.*[*^11^*](#_ENREF_11) | 175 | 168 | GG | - | - | - | - | - | - | - | - | - |
|  |  | 25 | 31 | GA | 0.774 | 0.439-1.366 | 0.377 | 0.756 | 0.434-1.319 | 0.325 | - | - | - |
|  |  | 1 | 2 | AA | 0.480 | 0.043-5.343 | 0.551 |  |  |  | 0.497 | 0.045-5.531 | 0.570 |
|  | *Combined |  |  | GG | - | - | - | - | - | - | - | - | - |
|  |  | 1419 | 1072 | GA | **1.086** | **0.861-1.369** | **0.313** | **1.064** | **0.846-1.339** | **0.346** | - | - | - |
|  |  |  |  | AA | **0.690** | **0.240-1.984** | **0.315** |  |  |  | **0.683** | **0.237-1.963** | **0.310** |
| rs1800469 | *Syrris et al.*[*^13^*](#_ENREF_13) | 301 | 124 | CC | - | - | - | - | - | - | - | - | - |
|  |  | 284 | 97 | CT | 1.206 | 0.884-1.647 | 0.238 | 1.215 | 0.905-1.631 | 0.194 | - | - | - |
|  |  | 70 | 23 | TT | 1.254 | 0.749-2.099 | 0.390 |  |  |  | 1.150 | 0.700-1.888 | 0.581 |
|  | *Cambien et al.*[*^20^*](#_ENREF_20) | 240 | 263 | CC | - | - | - | - | - | - | - | - | - |
|  |  | 257 | 297 | CT | 0.948 | 0.745-1.208 | 0.667 | 0.967 | 0.768-1.217 | 0.776 | - | - | - |
|  |  | 66 | 69 | TT | 1.048 | 0.717-1.533 | 0.808 |  |  |  | 1.078 | 0.753-1.542 | 0.682 |
|  | *Koch et al.*[*^19^*](#_ENREF_19) | 1581 | 564 | CC | - | - | - | - | - | - | - | - | - |
|  |  | 1659 | 508 | CT | 1.165 | 1.015-1.338 | 0.030 | 1.145 | 1.005-1.304 | 0.042 | - | - | - |
|  |  | 417 | 139 | TT | 1.070 | 0.863-1.326 | 0.536 |  |  |  | 0.993 | 0.809-1.217 | 0.943 |
|  | *Crobu et al.*[*^11^*](#_ENREF_11) | 67 | 80 | CC | - | - | - | - | - | - | - | - | - |
|  |  | 87 | 92 | CT | 1.129 | 0.729-1.749 | 0.586 | 1.322 | 0.880-1.987 | 0.179 | - | - | - |
|  |  | 47 | 29 | TT | 1.935 | 1.100-3.406 | 0.022 |  |  |  | 1.810 | 1.086-3.018 | 0.023 |
|  | *Combined |  |  | CC | - | - | - | - | - | - | - | - | - |
|  |  | 5076 | 2285 | CT | **1.120** | **1.005-1.249** | **0.049** | **1.125** | **1.016-1.247** | **0.031** | - | - | - |
|  |  |  |  | TT | **1.175** | **0.946-1.459** | **0.137** |  |  |  | **1.135** | **0.907-1.419** | **0.216** |
| rs1800470 | *Syrris et al.*[*^13^*](#_ENREF_13) | 242 | 102 | TT | - | - | - | - | - | - | - | - | - |
|  |  | 306 | 109 | TC | 1.183 | 0.861-1.627 | 0.300 | 1.226 | 0.908-1.654 | 0.183 | - | - | - |
|  |  | 107 | 33 | CC | 1.367 | 0.868-2.151 | 0.177 |  |  |  | 1.248 | 0.819-1.903 | 0.302 |
|  | *Cambien et al.*[*^20^*](#_ENREF_20) | 181 | 225 | TT | - | - | - | - | - | - | - | - | - |
|  |  | 277 | 297 | TC | 1.159 | 0.898-1.496 | 0.256 | 1.175 | 0.924-1.465 | 0.188 | - | - | - |
|  |  | 105 | 107 | CC | 1.220 | 0.874-1.702 | 0.242 |  |  |  | 1.118 | 0.831-1.505 | 0.460 |
|  | *Koch et al.*[*^19^*](#_ENREF_19) | 1235 | 458 | TT | - | - | - | - | - | - | - | - | - |
|  |  | 1802 | 565 | TC | 1.183 | 1.025-1.365 | 0.021 | 1.193 | 1.042-1.365 | 0.010 | - | - | - |
|  |  | 620 | 188 | CC | 1.223 | 1.006-1.487 | 0.043 |  |  |  | 1.111 | 0.930-1.327 | 0.247 |
|  | *Crobu et al.*[*^11^*](#_ENREF_11) | 55 | 69 | TT | - | - | - | - | - | - | - | - | - |
|  |  | 88 | 101 | TC | 1.093 | 0.693-1.723 | 0.702 | 1.388 | 0.907-2.123 | 0.131 | - | - | - |
|  |  | 58 | 31 | CC | 2.347 | 1.338-4.117 | 0.003 |  |  |  | 2.224 | 1.363-3.629 | 0.001 |
|  | *Yokota et al.*[*^18^*](#_ENREF_18) | 89 | 149 | TT | - | - | - | - | - | - | - | - | - |
|  |  | 185 | 295 | TC | 1.050 | 0.762-1.447 | 0.766 | 0.856 | 0.629-1.164 | 0.322 | - | - | - |
|  |  | 41 | 147 | CC | 0.467 | 0.302-0.721 | 0.001 |  |  |  | 0.452 | 0.310-0.659 | <0.001 |
|  | Holweg | 60 | 37 | TT | - | - | - | - | - | - | - | - | - |
|  |  | 70 | 43 | TC | 1.004 | 0.574-1.755 | 0.989 | 0.909 | 0.535-1.544 | 0.724 | - | - | - |
|  |  | 14 | 14 | CC | 0.617 | 0.264-1.438 | 0.263 |  |  |  | 0.615 | 0.279-1.358 | 0.229 |
|  | *Combined |  |  | TT | - | - | - | - | - | - | - | - | - |
|  |  | 5535 | 2970 | TC | **1.153** | **1.039-1.279** | **0.011** | **1.146** | **1.026-1.279** | **0.021** | - | - | - |
|  |  |  |  | CC | **1.082** | **0.743-1.575** | **0.367** |  |  |  | **1.013** | **0.452-1.464** | **0.398** |
| rs1800471 | *Syrris et al.*[*^13^*](#_ENREF_13) | 598 | 214 | GG | - | - | - | - | - | - | - | - | - |
|  |  | 95 | 30 | GC | 1.214 | 0.782-1.885 | 0.386 | 1.240 | 0.800-1.923 | 0.337 | - | - | - |
|  |  | 2 | 0 | CC | 1.920 | 0.092-40.163 | 0.674 |  |  |  | 1.871 | 0.089-39.104 | 0.686 |
|  | *Cambien et al.*[*^20^*](#_ENREF_20) | 464 | 546 | GG | - | - | - | - | - | - | - | - | - |
|  |  | 92 | 81 | GC | 1.337 | 0.967-1.847 | 0.079 | 1.404 | 1.022-1.927 | 0.036 | - | - | - |
|  |  | 7 | 2 | CC | 4.119 | 0.851-19.922 | 0.078 |  |  |  | 3.947 | 0.817-19.078 | 0.088 |
|  | *Koch et al.*[*^19^*](#_ENREF_19) | 3149 | 1063 | GG | - | - | - | - | - | - | - | - | - |
|  |  | 486 | 141 | GC | 1.164 | 0.953-1.421 | 0.138 | 1.159 | 0.953-1.409 | 0.140 | - | - | - |
|  |  | 22 | 7 | CC | 1.061 | 0.452-2.491 | 0.892 |  |  |  | 1.041 | 0.444-2.443 | 0.926 |
|  | *Holweg et al.*[*^12^*](#_ENREF_12) | 123 | 79 | GG | - | - | - | - | - | - | - | - | - |
|  |  | 18 | 15 | GC | 0.771 | 0.367-1.617 | 0.491 | 0.899 | 0.438-1.848 | 0.772 | - | - | - |
|  |  | 3 | 0 | CC | 4.506 | 0.230-88.410 | 0.322 |  |  |  | 4.675 | 0.239-91.544 | 0.310 |
|  | *Combined |  |  | GG | - | - | - | - | - | - | - | - | - |
|  |  | 5019 | 2178 | GC | **1.186** | **1.016-1.385** | **0.039** | **1.207** | **1.037-1.406** | **0.021** | - | - | - |
|  |  |  |  | CC | **1.563** | **0.770-3.171** | **0.186** |  |  |  | **1.530** | **0.754-3.105** | **0.199** |
| rs1800472 | *Syrris et al.*[*^13^*](#_ENREF_13) | 622 | 237 | CC | - | - | - | - | - | - | - | - | - |
|  |  | 33 | 7 | CT | 1.796 | 0.784-4.116 | 0.166 | 1.796 | 0.784-4.116 | 0.166 | - | - | - |
|  |  | 0 | 0 | TT | 0.382 | 0.008-19.283 | 0.630 |  |  |  | 0.373 | 0.007-18.850 | 0.622 |
|  | *Cambien et al.*[*^20^*](#_ENREF_20) | 563 | 585 | CC | - | - | - | - | - | - | - | - | - |
|  |  | 27 | 42 | CT | 0.668 | 0.406-1.098 | 0.112 | 0.638 | 0.389-1.044 | 0.074 | - | - | - |
|  |  | 0 | 2 | TT | 0.208 | 0.010-4.338 | 0.311 |  |  |  | 0.213 | 0.010-4.436 | 0.318 |
|  | *Koch et al.*[*^19^*](#_ENREF_19) | 3421 | 1138 | CC | - | - | - | - | - | - | - | - | - |
|  |  | 231 | 75 | CT | 1.025 | 0.783-1.341 | 0.860 | 1.033 | 0.791-1.349 | 0.812 | - | - | - |
|  |  | 5 | 1 | TT | 1.663 | 0.194-14.251 | 0.642 |  |  |  | 1.661 | 0.194-14.229 | 0.643 |
|  | *Combined |  |  | CC | - | - | - | - | - | - | - | - | - |
|  |  | 4902 | 2087 | CT | **0.985** | **0.647-1.499** | **0.398** | **0.978** | **0.622-1.536** | **0.397** | - | - | - |
|  |  |  |  | TT | **0.730** | **0.147-3.622** | **0.371** |  |  |  | **0.732** | **0.148-3.628** | **0.371** |

OR, odds ratio; CI, 95% confidence intervals; N, number of individuals. *Combined, indicates meta-analysis of data by random effects model.
