# Supplementary material for: Meta-Analysis of the Association between Transforming Growth Factor-Beta Polymorphisms and Complications of Coronary Heart Disease
Source: PLoS One. 2012 May 25;7(5):e37878. doi: 10.1371/journal.pone.0037878 (PMC3360665; doi:10.1371/journal.pone.0037878)
Supplement: Text S1 — Protocol used for systematic data collection from eligible articles. (DOC) [file pone.0037878.s004.doc]

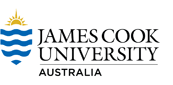


**Data Extraction Form**

Reviewer: Date:

Article Title: First Author:

Journal Title: Year of Publication:

1. **Study Design**

Is the objective of the study clearly stated in the abstract? Yes No

If so, what is it?

Is the study design clearly stated? Yes No

What is the study design?

Notes:

1. **Participants**

Is the sample size clearly stated? Yes No

State the sample size. N =

What are the different population groups? (e.g. cases, controls)
State the number of participants in each group.

Group 1: N= Group 2: N=

Group 3: N= Group 4: N=

More groups:

Are the methods for recruitment and sampling clearly stated? Yes No

If so, how were the patients recruited?

If possible, state where the patients were recruited from:

Is one or more of the sample populations defined by an end stage complication of CHD?

Yes No

If so, which?

Myocardial Infarction Sudden Cardiac Death Angina Heart Failure

Was the control group screened for CHD (if applicable)? Yes No Not stated

If so, what was the method of screening?

Coronary angiography Not stated Other:

Is the inclusion and exclusion criteria stated? Yes No

Inclusion criteria:

Exclusion criteria

Notes:

1. **Population Characteristics**

What is the age range of each population?

Group 1: Mean Range

Group2: Mean Range

Group3: Mean Range

Group4: Mean Range

Is this a potential source of bias? Yes No Unclear

What is the percentage of males in each group?

Group 1: Group 2: Group 3: Group 4:

Is this a potential source of bias? Yes No Unclear

Did the study account for other risk factors? Yes No

Smoking Diabetes Dyslipidaemia Hypertension Family history

For other risk factors that were discussed, fill in the following table:

|  | Group 1 | Group 2 | Group 3 | Group 4 |
| --- | --- | --- | --- | --- |
| Family history |  |  |  |  |
| Hypertension |  |  |  |  |
| Diabetes |  |  |  |  |
| Dyslipidaemia |  |  |  |  |
| Smokers |  |  |  |  |

Notes:

1. **Genetics**

Which of the following genes did the study focus on?

TGF-β1 TGF-β2 TGF-β3

TGFBR1 TGFBR2

What nomenclature was used for the description of SNPs? Give an example.

rs number amino acid sequence alteration DNA sequence alteration

Other Example:

List all SNPs assessed, relevant to the above genes:

What laboratory method was used to assess SNP frequency?

1. **Results**

What is the frequency of SNPs in the different population groups?

| SNP allele | Group 1 frequency | Group 2 frequency | Group 3 frequency | Group 4 frequency |
| --- | --- | --- | --- | --- |
|  |  |  |  |  |
|  |  |  |  |  |
|  |  |  |  |  |
|  |  |  |  |  |
|  |  |  |  |  |
|  |  |  |  |  |
|  |  |  |  |  |
|  |  |  |  |  |
|  |  |  |  |  |
|  |  |  |  |  |
|  |  |  |  |  |
|  |  |  |  |  |
|  |  |  |  |  |
|  |  |  |  |  |
|  |  |  |  |  |

For results that are statistically significant, list the relevant SNP, odds ratio, C.I. and p value:

For results that are not statistically significant, list the relevant SNP, odds ratio, C.I. and p value:

1. **Discussion and Conclusion**

What limitations were identified in the study by the author?

What conclusions were made about the SNPs assessed and the disease of interest?
